# Validation of an automated technique for ovarian cortex dissociation: isolation of viable ovarian cells and their qualification by multicolor flow cytometry

**DOI:** 10.1186/s13048-017-0337-0

**Published:** 2017-06-23

**Authors:** Tristan Zver, Elodie Mouloungui, Aurélie Berdin, Christophe Roux, Clotilde Amiot

**Affiliations:** 1 Centre Hospitalier Régional Universitaire Jean Minjoz, Service de Biologie et Médecine de la Reproduction - Cryobiologie, CECOS Franche-Comté Bourgogne, CIC-1431, 3 boulevard Fleming, F-25030 Besançon Cedex, France; 2 INSERM UMR 1098, 8 rue du Docteur Jean-François-Xavier Girod, BP1937, F-25020 Besançon Cedex, France; 3 Université de Franche-Comté, 1 rue Goudimel, F-25030 Besançon Cedex, France; 4 Centre Hospitalier Régional Universitaire Jean Minjoz, Service de Gynécologie-obstétrique, 3 boulevard Fleming, F-25030 Besançon Cedex, France

**Keywords:** Fertility preservation, Ovarian tissue, Tissue dissociation, Cell qualification, Multicolor flow cytometry

## Abstract

**Background:**

Ovarian tissue cryopreservation is a technique for fertility preservation addressed to prepubertal girls or to patients for whom no ovarian stimulation is possible before initiation of gonadotoxic treatments. Autotransplantation of frozen-thawed ovarian tissue is the only available option for reuse but presents some limitations: ischemic tissue damages post-transplant and reintroduction of malignant cells in cases of cancer. It is therefore essential to qualify ovarian tissue before autograft on a functional and oncological point of view. Here, we aimed to isolate viable cells from human ovarian cortex in order to obtain an ovarian cell suspension analyzable by multicolor flow cytometry.

**Methods:**

Ovarian tissue (fresh or frozen-thawed), from patients with polycystic ovarian syndrome (reference tissue) and from patients who underwent ovarian tissue cryopreservation, was used for dissociation with an automated device. Ovarian tissue-dissociated cells were analyzed by multicolor flow cytometry; the cell dissociation yield and viability were assessed. Two automated dissociation protocols (named laboratory and commercial protocols) were compared.

**Results:**

The effectiveness of the dissociation was not significantly different between reference ovarian tissue (1.58 × 10^6^ ± 0.94 × 10^6^ viable ovarian cells per 100 mg of ovarian cortex, *n* = 60) and tissue from ovarian tissue cryopreservation (1.70 × 10^6^ ± 1.35 × 10^6^ viable ovarian cells, *n* = 18). However, the viability was slightly different for fresh ovarian cortex compared to frozen-thawed ovarian cortex whether we used reference tissue (*p* = 0.022) or tissue from ovarian cryopreservation (*p* = 0.018). Comparing laboratory and commercial protocols, it appeared that cell yield was similar but cell viability was significantly improved when using the commercial protocol (81.3% ± 12.3% vs 23.9% ± 12.5%).

**Conclusion:**

Both dissociation protocols allow us to isolate more than one million viable cells per 100 mg of ovarian cortex, but the viability is higher when using the commercial dissociation kit. Ovarian cortex dissociation is a promising tool for human ovarian cell qualification and for ovarian residual disease detection by multicolor flow cytometry.

## Background

Although cancer remains one of the most important causes of mortality, therapeutic advances have allowed a huge increase of the survival during these last years. In Europe, overall survival at 5 years reaches 78.3% and 71.9% for girls (0-15) and women (15-44) diagnosed between 2000 and 2007 respectively [1, 2]. Unfortunately, some chemotherapy and/or radiotherapy regimens are highly gonadotoxic and could induce premature ovarian failure.

Women with cancer have several options to preserve their fertility: ovarian transposition (only in cases of pelvic irradiation), embryo or oocyte cryopreservation and ovarian tissue cryopreservation (OTC) [3, 4] (which can be combined with immature oocytes collection [5, 6]). Currently, embryo and oocyte cryopreservation are the only established methods endorsed by the American Society of Reproductive Medicine (ASRM) [7], the American Society of Clinical Oncology (ASCO) [8] and the European Society for Medical Oncology (ESMO) [9]. Ovarian cortex cryopreservation is recognized in France as one fertility preservation option (law of Bioethics n°2004–800) and has several advantages as no ovarian stimulation is required and it can be proposed to prepubertal girls or patients in whom gonadotoxic treatment cannot be postponed [10].

Ovarian tissue reuse is still considered experimental, while some authors suggest that autotransplantation of frozen-thawed ovarian tissue should now be considered as established procedure for female fertility preservation [11–13]. Indeed, transplantation of frozen-thawed ovarian tissue has already resulted in 86 reported live births worldwide [14, 15] and, in a recent study including 111 women, the proportion of women who conceived after autograft of cryopreserved ovarian tissue was 29% [11]. Nevertheless, this technique presents some limitations: especially ischemic tissue damages after ovarian tissue transplantation, which lead to follicular loss [16–19]; and the risk of reintroducing malignant cells in cases of malignancies that may metastasize to the ovary.

Some studies have shown that malignant cells could be identified in ovarian tissue by using real-time quantitative polymerase chain reaction (RT-qPCR), human ovarian tissue xenografts into immunodeficient mice [20–22] or multicolor flow cytometry (MFC) as demonstrated previously by our team [23–25]. MFC allowed us to differentiate and quantify viable leukemic cells among viable human ovarian cells. It is therefore important to obtain an ovarian cell suspension after ovarian tissue dissociation that can be analyzed by MFC.

The aim of the current study is to validate an automated dissociation technique, combining mechanical and enzymatic effects, in order to obtain ovarian cortex tissue-dissociated cells, especially somatic cells such as stromal extravascular cells and endothelial cells. We used two different sources of human ovarian tissue: reference tissue collected during ovarian drilling and tissue from patients who underwent OTC. The ovarian suspension was analyzed by MFC to identify viable ovarian cells and to determine the cell viability rate after ovarian tissue dissociation. Finally, we compared our dissociation method (named laboratory protocol) with a commercial dissociation kit in order to try and improve ovarian tissue dissociation.

## Methods

### Ovarian tissue samples

**Fig. 1:**
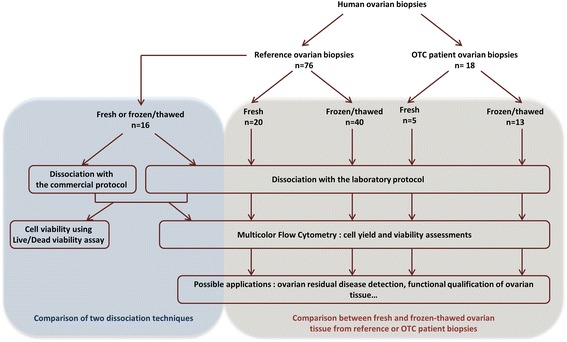
Experimental design

The experimental design of the study is shown in Fig. 1. Reference ovarian tissue samples, commonly used in the laboratory, from women undergoing laparoscopic drilling for polycystic ovary syndrome (PCOS, 23-38 years of age, *n* = 76) and ovarian cortical tissue from patients in whom OTC was performed for different pathologies (6-33 years of age, *n* = 18: acute leukemia *n* = 13, Ewing’s sarcoma *n* = 2, Hodgkin’s lymphoma *n* = 2, and systemic lupus erythematosus *n* = 1), were used to validate the ovarian tissue dissociation method. All patients received chemotherapy before OTC.

### Freezing-thawing and isolation procedure for ovarian tissue

Cortical biopsies were used, either fresh (*n* = 25) or after cryopreservation (*n* = 53), according to a protocol using slow cooling with manual seeding [26]. After freezing, the vials were stored in liquid nitrogen. Ovarian cortical biopsies were thawed according to the technique previously described [27].

Fragments used for this study were weighed (106.4 mg ± 63.4 [range = 20.9-276.4], *n* = 60 for reference ovarian tissue; 180.8 mg ± 228.8 [range = 54.0-1070.0], *n* = 18 for ovarian tissue from OTC) to determine the cell dissociation yield and then compare cell yield between each ovarian tissue sample. Pieces of ovarian cortex, either fresh or frozen-thawed, underwent a mechanical and enzymatic dissociation using an automated cell dissociator (gentleMACS™ Dissociator, Miltenyi Biotec SAS, Paris, France) after being sectioned into pieces of ≈ 1-2 mm^3^ [23]. The isolation procedure for ovarian cells developed in our laboratory is based on enzymatic dissociation by collagenase Ia (100 mg/mL; Sigma; Saint-Quentin Fallavier, France) and DNase I (0.1 mg/mL; Roche Diagnosis, Meylan, France) in 5 mL of RPMI (PAA laboratories, Les Mureaux, France) using C Tubes (Miltenyi Biotec SAS) for 40 min at 37 °C under gentle agitation. After ovarian tissue dissociation, we performed filtration with a 70 μm cell strainer (Dutscher SAS, Brumath, France) to eliminate the residual connective tissue fibres and washed with 5 mL of RPMI. The cell suspension was centrifuged at 300 g for 7 min at 4 °C and the pellet was resuspended in an appropriate volume of RPMI.

We also tested a commercial dissociation kit using the same automated cell dissociator (Tumor Dissociation Kit, human ref. 130-095-929, Miltenyi Biotec), but differing from the laboratory protocol regarding the enzymes used (3 different enzymes called H, R and A) and the time of incubation at 37 °C (one hour). We compared the quality of ovarian tissue dissociation between both isolation procedures.

In a previous article, we demonstrated that the freezing-thawing process as well as the enzymatic procedure carried out in our laboratory protocol had no effect on the expression of cell surface markers used in this study [23]. The test performed by the manufacturer for the commercial kit are in accordance with these findings.

### Ovarian cell suspension analysis by multicolor flow cytometry

**Fig. 2:**
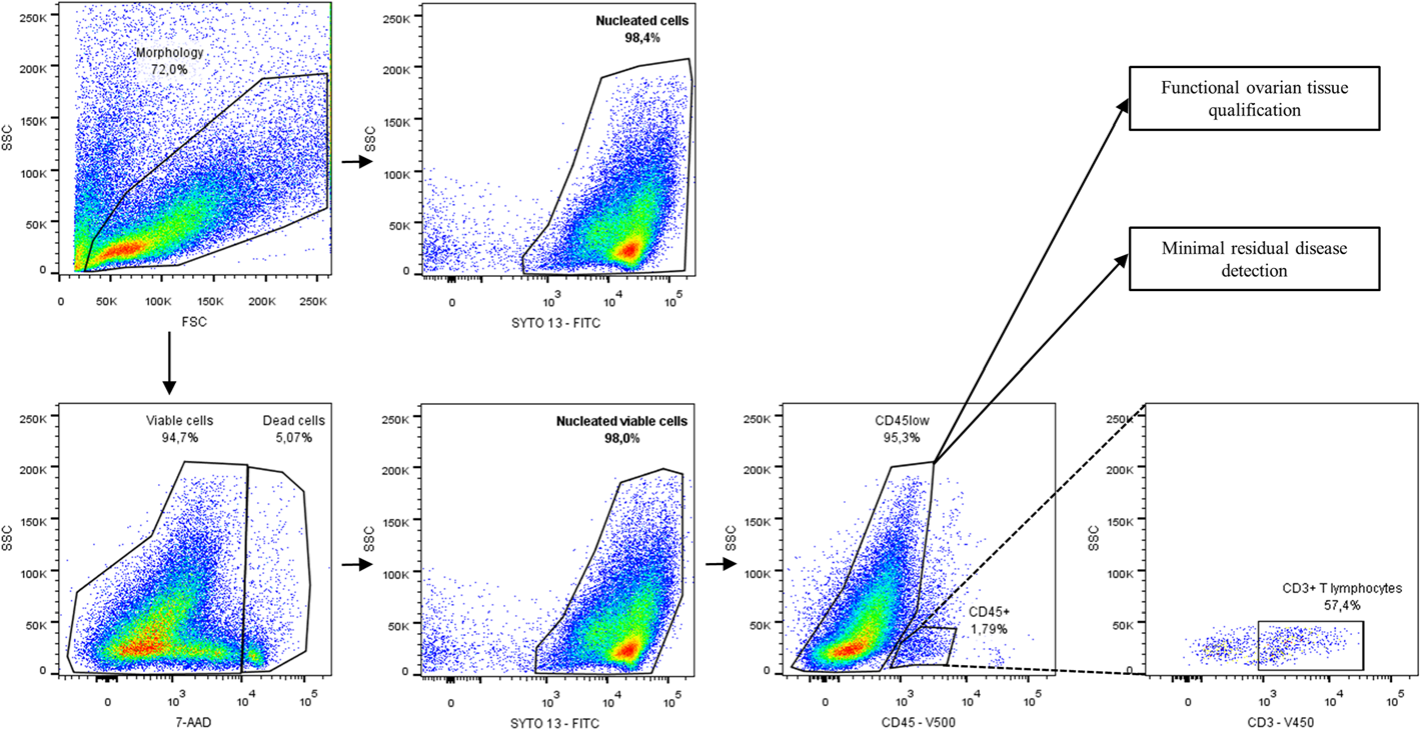
MFC gating strategy applied to all ovarian cell suspensions. The first morphological gate is used for debris exclusion using SSC and FSC light scatter (Morphology). Then, an additional gating was performed using SYTO 13 to set nucleated cells (SYTO13^+^) and 7-AAD^−^/SYTO13^+^combination to identify nucleated viable cells. The populations in bold were used to assess cell viability after ovarian tissue dissociation. CD45 gating was used to exclude CD45^+^/CD3^+^ normal T lymphocytes and to select CD45^low^ population for further functional ovarian tissue qualification or minimal residual disease detection

MFC was performed using a BD CANTO II flow cytometer and FACSDiva software (BD Biosciences, Franklin Lakes, NJ, USA). Instrument settings were in accordance with standardized Euroflow protocols [28]. The compensation matrix was set up as previously described [23]. The antibody panel included 7-Amino-Actinomycin D (7-AAD) (Beckman Coulter, Fullerton, CA, USA), SYTO 13 (Invitrogen, Carlsbad, CA, USA), CD45 coupled with Horizon V500 (V500) (HI30, BD Biosciences, Franklin Lakes, NJ, USA) and CD3 coupled with Horizon V450 (V450) (UCHT1, BD Biosciences). At least 10,000 total events were acquired for the analysis. Gating strategy (Fig. 2) was based on the elimination of debris by an initial morphological gate using forward (FSC) and side (SSC) light scatter characteristics. Nucleated viable cells were then selected by their SYTO 13^+^/7-AAD^−^ phenotype. Within these cells, we identified CD45^+^ and CD3^+^ T lymphocytes. Cell yield is given as the number of viable ovarian cells per 100 mg of dissociated tissue, Flowcount™ Fluorospheres (Beckman Coulter, Fullerton, CA, USA) being used for absolute count. Viability is equal to the ratio between the number of viable nucleated events and the number of nucleated events (Fig. 2).

### Observation of ovarian cell suspension viability by fluorescence microscopy

Cell viability was assessed by using Live/Dead assay kit as described in the manufacturer’s protocol (LIVE/DEAD® Viability/Cytotoxicity Kit for mammalian cells, Molecular Probes™, Life Technologies SAS, Eugene, USA). Briefly, ovarian cells were incubated in 10 mL PBS containing 4 mM Calcein AM and 2 mM Ethidium homodimer-1 (EthD-1) for 30 min at 25 °C in the dark. After exposure to fluorescent dyes, ovarian cells were observed under an inverted fluorescence microscope (CKX41, Olympus France SA) equipped with a CCD Color Peltier Cooled camera (Moticam Pro 282B, Motic, Hong Kong). Viable isolated ovarian cells are stained with Calcein AM which emits green fluorescence (517 nm) when excited by blue light (494 nm); whereas dead cells are stained by EthD-1, which emits red fluorescence (617 nm) when excited by green light (528 nm).

### Statistical analysis

Using the Mann-Whitney test, we compared cell yield and viability from reference ovarian tissue and patient ovarian samples, both fresh and frozen-thawed. A Wilcoxon match-pairs signed rank test was used to compare cell yield and viability results between the two different dissociation protocols. Results were plotted using GraphPad software (GraphPad Software Inc., San Diego, CA, USA). A *p*-value of less than 0.05 was considered statistically significant for all tests.

## Results

### Assessment by multicolor flow cytometry of isolated viable cells obtained from ovarian cortex

The ovarian cell suspension obtained after ovarian cortex dissociation was analyzed by MFC to quantify viable nucleated cells (SYTO 13^+^/7-AAD^−^) and to determine the cell yield of the dissociation technique (results extrapolated to 100 mg of ovarian tissue).

Table 1 shows the results of the cell yield and viability obtained by MFC after dissociation of reference ovarian tissue or tissue from OTC.

**Table 1:** Cell yield and viability obtained by multicolor flow cytometry analysis after dissociation of reference ovarian tissue and tissue for ovarian tissue cryopreservation

Parameters	Yield (living ovarian cells/100 mg of tissue)				Viability (percentage)			
Tissue origin	Reference ovarian tissue		Tissue from OTC		Reference ovarian tissue		Tissue from OTC	
Fresh or frozen-thawed	Fresh	Frozen-thawed	Fresh	Frozen-thawed	Fresh	Frozen-thawed	Fresh	Frozen-thawed
n	20	40	5	13	20	40	5	13
Mean	1.56 × 10^6^	1.59 × 10^6^	3.31 × 10^6^	1.09 × 10^6^	38.2	27.0	42.4	16.9
Standard deviation	0.94 × 10^6^	1.35 × 10^6^	2.41 × 10^6^	0.64 × 10^6^	18.2	15.4	21.4	14.3
Minimum	0.34 × 10^6^	0.20 × 10^6^	0.25 × 10^6^	0.81 × 10^5^	9.0	2.0	17.0	1.0
Maximum	4.28 × 10^6^	6.85 × 10^6^	6.22 × 10^6^	2.33 × 10^6^	73.0	67.0	67.0	51.0

### Cell yield and viability comparison between reference ovarian tissue and patient samples from OTC by multicolor flow cytometry

**Fig. 3:**
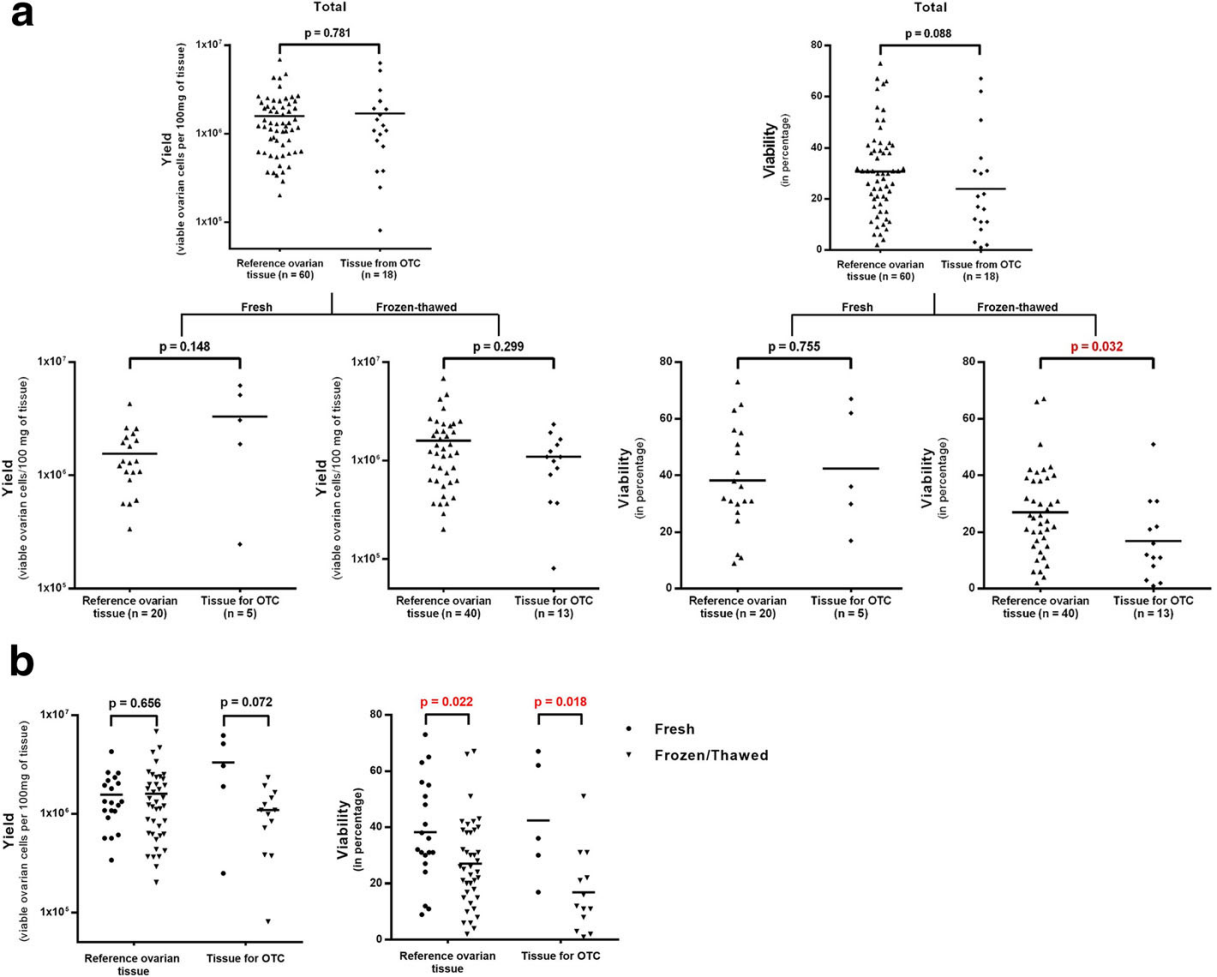
Assessment of cell yield and viability by multicolor flow cytometry after ovarian tissue dissociation. **a** Comparison between reference ovarian tissue and tissue from patients undergoing ovarian tissue cryopreservation in terms of yield and viability. **b** Comparison between fresh and frozen-thawed ovarian tissue in terms of yield and viability. The mean value for each data set is represented by a horizontal bar. Significant *p*-values are indicated in red. OTC - ovarian tissue cryopreservation

No statistically significant difference was observed for cell yield between reference ovarian tissue (1.58 × 10^6^ viable cells per 100 mg of tissue ± 1.22 × 10^6^, *n* = 60) and tissue from OTC (1.70 × 10^6^ viable cells per 100 mg of tissue ± 1.65 × 10^6^, *n* = 18) (*p* = 0.781, Fig. 3a). Then we analyzed results either with fresh or frozen-thawed ovarian tissue. For both types of ovarian tissue, there is no difference in cell yield depending on whether we used fresh (*p* = 0.148) or frozen-thawed (*p* = 0.299) ovarian tissue (Fig. 3a).

No significant difference was also observed for cell viability between the two types of ovarian tissue after dissociation: 31% for reference ovarian tissue (*n* = 60) and 24% for OTC tissue (*n* = 18) (*p* = 0.088) (Fig. 3a). However, looking specifically at fresh and frozen-thawed tissues, we saw no difference for fresh ovarian tissue (*p* = 0.755) and a slight difference between frozen-thawed reference ovarian tissue and frozen-thawed tissue from OTC (*p* = 0.032) (Fig. 3a).

### Cell yield and viability comparison between fresh and frozen-thawed ovarian tissue by multicolor flow cytometry

Cell yield and viability results were also analyzed regarding the potential impact of cryopreservation on reference ovarian tissue or tissue from OTC.

There was no significant difference in cell yield whether the tissue was fresh or frozen-thawed (reference tissue: *p* = 0.656; tissue for OTC: *p* = 0.072). However, there was a significant difference in cell viability between fresh and frozen-thawed tissue (reference tissue: *p* = 0.022; tissue for OTC: *p* = 0.018). Indeed, ovarian cell viability is increased when the tissue is fresh rather than frozen-thawed (Fig. 3b).

### Comparison between laboratory and commercial dissociation methods

**Fig. 4:**
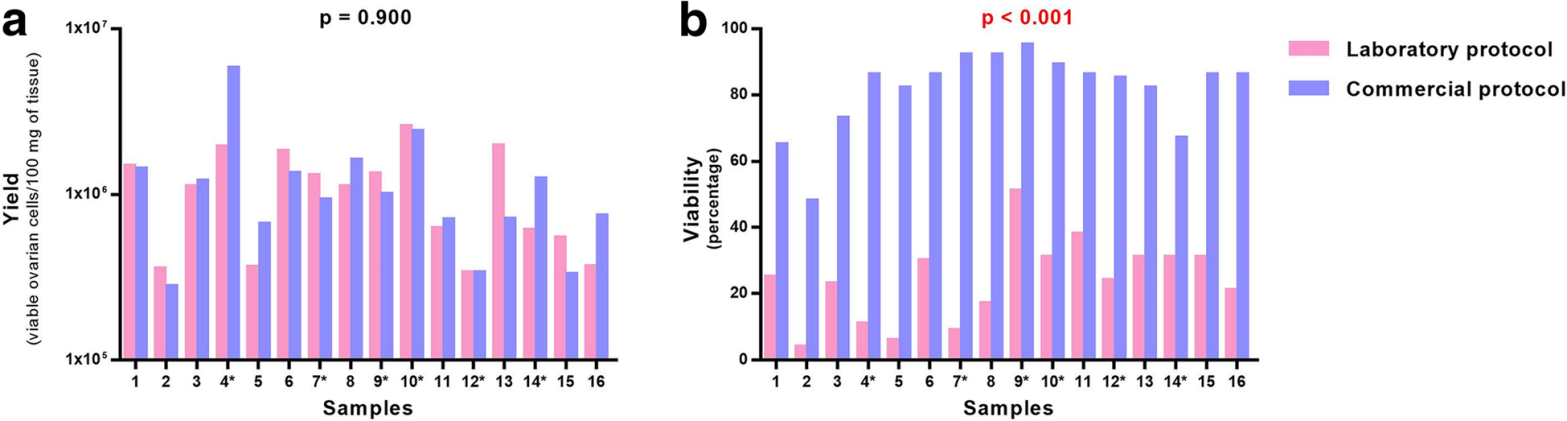
Comparison between laboratory and commercial protocol for ovarian tissue dissociation. Results are expressed in terms of cell yield (**a**) and cell viability (**b**). Samples with an asterisk (*) correspond to fresh ovarian samples (*n* = 5). Significant *p*-values are indicated in *red*

Using MFC analysis, we compared ovarian cell yield and viability obtained after fresh or frozen-thawed reference ovarian tissue dissociation (16 different samples) performed with our laboratory protocol or with the commercial protocol (Fig. 4).

The cell yield was not significantly different between the laboratory protocol (1.18 × 10^6^ ± 0.71 × 10^6^ viable nucleated cells per 100 mg of reference ovarian tissue [range = 0.34 × 10^6^-2.59 × 10^6^], *n* = 16) and the commercial protocol (1.29 × 10^6^ ± 1.31 × 10^6^ [range = 0.28 × 10^6^-5.76 × 10^6^], *n* = 16) (Fig. 4a). On the contrary, a significant decrease in viability was observed (*p* < 0.0001) with the laboratory protocol (23.9 ± 12.5% [range = 4-51], *n* = 16) in comparison to commercial protocol (81.3 ± 12.3% [range = 48-95], *n* = 16) (Fig. 4b).

**Fig. 5:**
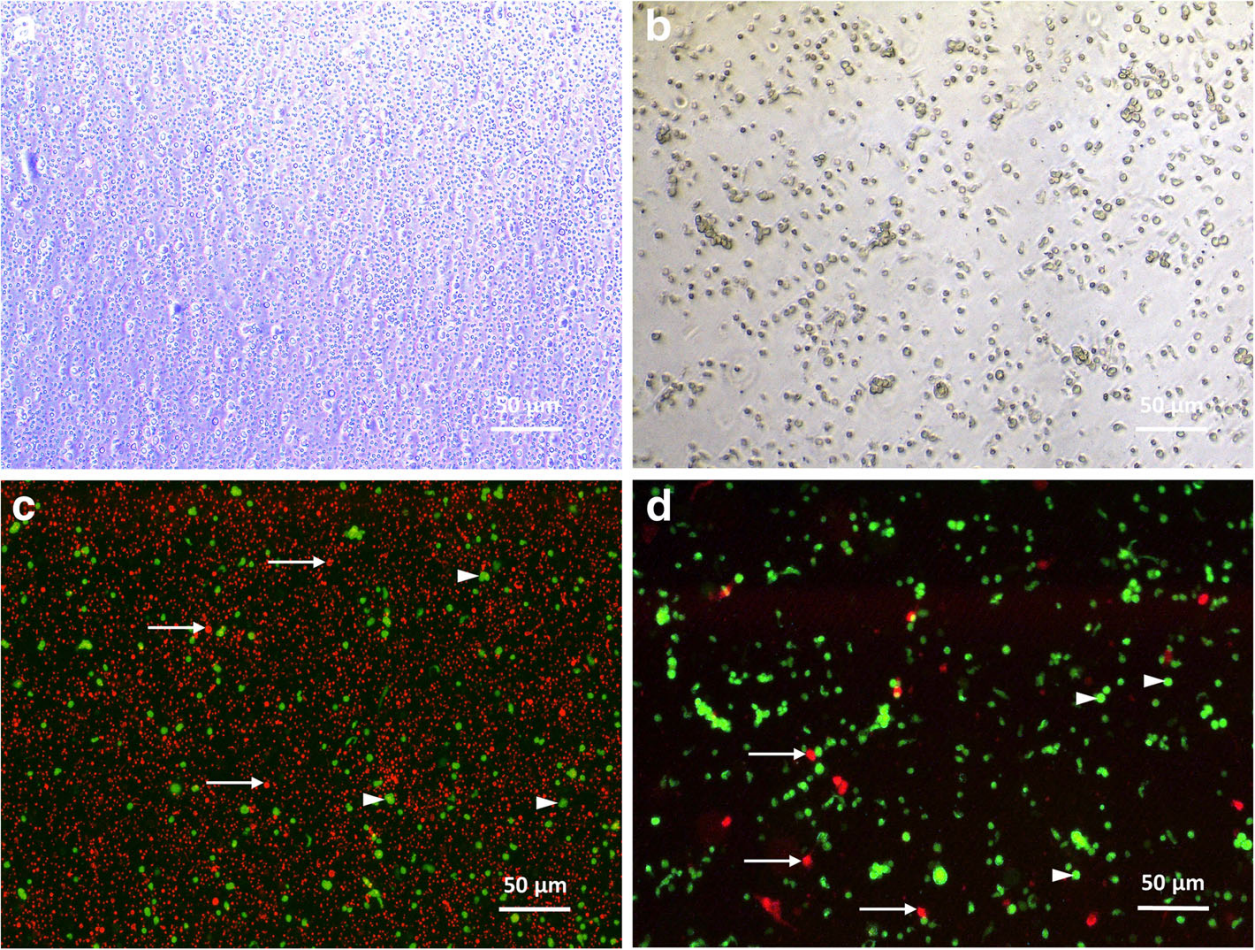
Ovarian single-cell suspension observed under light and fluorescent microscopes. Ovarian cells were observed with phase contrast microscopy after reference ovarian tissue dissociation using laboratory (**a**) or commercial (**b**) protocols. Merged images (**c**, **d**) show expression of Calcein AM (in *green*: viable cells - *arrowheads*) and ethidium homodimer-1 (in *red*: dead cells - *arrows*, and debris)

Light microscope observation of ovarian cell suspensions revealed less debris when using the commercial protocol (Fig. 5a and b). Fluorescence microscopy showed a higher number of dead cells (in red) when using the laboratory dissociation protocol (Fig. 5c and d).

## Discussion

In this study, an original technique to isolate viable cells from human ovarian cortex was validated. Previous dissociation methods have been described in the literature: they’ve been used to isolate follicles from ovarian cortex [29–32] or discarded medulla tissue from women undergoing fertility preservation [33, 34]. The current study does not aspire to isolate ovarian follicles, but it aims to get an analyzable ovarian cell suspension for subsequent analysis by MFC.

Laboratory and commercial protocols used mechanical and enzymatic dissociation which allowed us to get a cell suspension analyzable by MFC. Although the laboratory dissociation protocol was previously tested by our team [23], we demonstrated here that the average viable ovarian cell yield obtained after ovarian tissue dissociation was higher than one million per 100 mg of tissue with both protocols. It can also suggest that an amount of 100 mg of ovarian tissue is sufficient to detect ovarian residual disease by MFC with a sensitivity of 10^−4^ [24, 25]. No difference was observed in terms of cell yield between reference ovarian tissue and tissue from OTC. This result demonstrates that our model of reference ovarian tissue obtained from women with PCOS is close to ovarian tissue from OTC.

Comparison between fresh and frozen-thawed ovarian tissue is important to demonstrate whether freezing-thawing procedure had an impact or not on cell yield and viability. The results obtained in this study have shown no difference in cell yield between fresh or frozen-thawed ovarian tissue. On the other hand, freezing leads to a slight decrease in viability after ovarian tissue thawing and dissociation. These results hold for reference ovarian tissue and cortex from OTC. Previous reports have suggested that slow freezing could have a negative impact on ovarian cells [35, 36]. Similar results were observed in our study, with decreased cell viability but no impact of slow freezing on cell yield as seen in Soares et al. [36]. This might be explained by the ovarian heterogeneity between different biopsies and patient samples. However, these findings are in agreement with the fact that the use of fresh or frozen-thawed ovarian tissue from the same patient should not change the results obtained after dissociation, which is very useful as ovarian minimal residual disease detection is most often performed on cryopreserved ovarian tissue.

All patients who underwent OTC had received chemotherapy before fertility preservation. Our results of ovarian tissue dissociation showed no difference in cell yield and viability between reference tissue and tissue for OTC. Therefore, chemotherapy seems to have no impact or a limited impact on the amount and viability of ovarian tissue-dissociated cells. This result is particularly interesting for ovarian residual disease detection as leukemia patients could have received chemotherapy prior to OTC: we won’t need a large number of ovarian cortical strips to obtain sufficient viable cells to perform ovarian residual disease with a robust sensitivity of 10^−4^ in ovarian cell suspension [24] as in blood or bone marrow [37]. However, the application of this dissociation technique to a larger number of ovarian tissues from patients undergoing OTC should allow to corroborate of this trend.

In the last part of this work, the laboratory protocol and the commercial kit were compared. A higher cell viability was observed using the commercial kit: this result is reflected by the reduction of cell debris observed by MFC after ovarian tissue dissociation. The tissue dissociation may be less traumatic for ovarian cells when using the commercial protocol. This MFC result was confirmed by assessing cell viability using fluorescent microscopy: less debris was observed after dissociation. And viable isolated ovarian follicles were identified after dissociation. Both dissociation protocols can be potentially used to isolate, in non-optimal conditions, primordial/primary ovarian follicles instead of the technique previously published for assessment of isolated follicle viability by trypan blue [26].

As described by our team, MFC analysis of the ovarian tissue-dissociated cells can identify malignant cells among viable ovarian cells [23–25]. Ovarian tissue-dissociated cells have the advantage of being analyzable by MFC and RT-qPCR using the same original sample [23]. In the frame of human ovarian tissue qualification, ovarian tissue dissociation and MFC can also be used together to identify ovarian cell subpopulations like CD34 or CD31-positive cells. These cells have been detected by immunohistochemistry and seem to be good predictors for neovascularization after ovarian tissue autotransplantation [38, 39]. Non-follicular ovarian cells might be assessed as a potential prognostic factor of ovarian function recovery in case of cryopreserved ovarian cortex re-use.

## Conclusions

The dissociation protocols proposed in this study are essential to obtain an ovarian cell suspension before MFC analysis. The commercial kit improves viability after dissociation, is easy to use and facilitates MFC analysis of ovarian cell suspensions. MFC appears to be a good mean for minimal residual disease research and human ovarian tissue qualification before autograft. From our point of view, a controlled dissociation protocol associated to MFC is a promising tool for ovarian tissue qualification and minimal residual detection in human ovarian cortex.

## Abbreviations

EthD-1: Ethidium homodimer-1
FSC: Forward light scatter
MFC: Multicolor flow cytometry
OTC: Ovarian tissue cryopreservation
PCOS: Polycystic ovary syndrome
RPMI: Roswell park memorial institute medium
RT-qPCR: Real-time quantitative polymerase chain reaction
SSC: Side light scatter
